# RCEAU-Net: Cascade Multi-Scale Convolution and Attention-Mechanism-Based Network for Laser Beam Target Image Segmentation with Complex Background in Coal Mine

**DOI:** 10.3390/s24082552

**Published:** 2024-04-16

**Authors:** Wenjuan Yang, Yanqun Wang, Xuhui Zhang, Le Zhu, Zhiteng Ren, Yang Ji, Long Li, Yanbin Xie

**Affiliations:** 1 School of Mechanical Engineering, Xi’an University of Science and Technology, No. 58, Mid-Yanta Road, Xi’an 710054, China; yangwenjuan@xust.edu.cn (W.Y.); 22205016033@stu.xust.edu.cn (Y.W.); 17691391172@163.com (L.Z.); 17829648401@163.com (Z.R.); wicked1997@163.com (Y.J.); 23205016032@stu.xust.edu.cn (L.L.); 15809297059@163.com (Y.X.); 2 Shaanxi Key Laboratory of Mine Electromechanical Equipment Intelligent Detection and Control, No. 58, Yanta Road, Xi’an 710054, China

**Keywords:** visual localization, U-Net, laser beams, image segmentation, RCEAU-Net

## Abstract

Accurate and reliable pose estimation of boom-type roadheaders is the key to the forming quality of the tunneling face in coal mines, which is of great importance to improve tunneling efficiency and ensure the safety of coal mine production. The multi-laser-beam target-based visual localization method is an effective way to realize accurate and reliable pose estimation of a roadheader body. However, the complex background interference in coal mines brings great challenges to the stable and accurate segmentation and extraction of laser beam features, which has become the main problem faced by the long-distance visual positioning method of underground equipment. In this paper, a semantic segmentation network for underground laser beams in coal mines, RCEAU-Net, is proposed based on U-Net. The network introduces residual connections in the convolution of the encoder and decoder parts, which effectively fuses the underlying feature information and improves the gradient circulation performance of the network. At the same time, by introducing cascade multi-scale convolution in the skipping connection section, which compensates for the lack of contextual semantic information in U-Net and improves the segmentation effect of the network model on tiny laser beams at long distance. Finally, the introduction of an efficient multi-scale attention module with cross-spatial learning in the encoder enhances the feature extraction capability of the network. Furthermore, the laser beam target dataset (LBTD) is constructed based on laser beam target images collected from several coal mines, and the proposed RCEAU-Net model is then tested and verified. The experimental results show that, compared with the original U-Net, RCEAU-Net can ensure the real-time performance of laser beam segmentation while increasing the Accuracy by 0.19%, Precision by 2.53%, Recall by 22.01%, and Intersection and Union Ratio by 8.48%, which can meet the requirements of multi-laser-beam feature segmentation and extraction under complex backgrounds in coal mines, so as to further ensure the accuracy and stability of long-distance visual positioning for boom-type roadheaders and ensure the safe production in the working face.

## 1. Introduction

Coal will remain the dominant energy source worldwide for decades to come. The automation of underground coal excavation processes has long been the focus of both industrial and academic research efforts. As the core equipment of the coal mine production system, the roadheader’s accurate pose estimation can improve the efficiency of roadway digging, and anticipate and avoid the collision of the roadheader with roadway support equipment in the complex geological environment, which is beneficial to reduce the risk of accidents, and ensure the safe and efficient production of the underground working face in coal mines [1].

At present, the position measurement technology of roadheading equipment includes inertial navigation and positioning technology, iGPS technology, total station measurement technology, ultra-wideband wireless communication technology (UWB) technology, machine vision measurement technology, etc. Among them, the inertial navigation positioning technology is less affected by environmental factors, but it is prone to cumulative errors during operation, and with the continuous operation in the working face, there are certain difficulties in the precise positioning of the roadheading equipment. Positioning based on iGPS is consistent with the GPS positioning method, which uses the triangulation principle to complete the spatial positioning of the measured point, but a large number of positioning signal transmitters will cause mutual obstruction of the measurement path and interference between the laser signals, and it is difficult to calibrate and install. At the same time, the accuracy of the receiver’s light sensitivity will also limit its positioning accuracy. The total station positioning method mainly calculates the position information under the coordinate system of the body of the roadheader based on the on-site arrangement of the coal mine equipment, but the underground environment of the coal mine is complex and variable, and it is necessary to combine a variety of feature datums to carry out the local coordinate transformation to obtain the accurate displacement value and to carry out the attitude estimation and the trajectory tracking in order to obtain the accurate position of the equipment, which makes the system complexity high. UWB-based measurement technology relies on the Time-of-Flight (TOF) and Time-Difference-of-Arrival (TDOA) principles to measure the time difference of the signals between the two ultra-wideband modules, so as to calculate the relative distance between the roadheader and the laser, and to complete the position measurement of the roadheader; however, due to the influence of the cut-off dust and occlusion on the digging surface, the UWB positioning error cannot meet the precise positioning requirements of the roadheader. The measurement information obtained by vision-based position measurement technology is intuitive and reliable, usually using visible light as the visual positioning target, and the spatial position of the body of the roadheader is obtained by adopting the target feature extraction and analyzing the relationship between the feature datum position and the roadway coordinate system, which has the advantages of non-contact, high measurement accuracy, low cost, and no accumulation of error, etc. Therefore, visual positioning is the most widely used positioning technology in coal mines.

**Figure 1:**
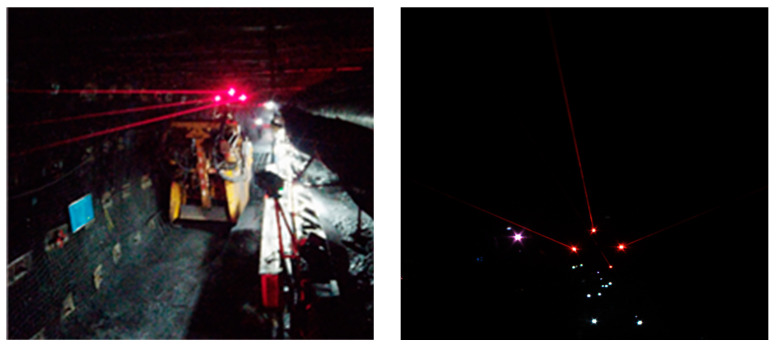
The laser beam-based visual positioning system that was under industrial testing at the tunneling face and its collected laser beam image.

In the previous work, we conducted a lot of research on the long-distance accurate pose estimation of the boom-type roadheader body, and developed a visual positioning system based on a laser beam target [2,3]. The method mainly relies on an industrial camera to collect the laser beam images formed by the laser pointing instrument to complete the remote distance positioning of the tunneling equipment. However, due to the interference of the harsh environment such as dust, water fog, and uneven illumination in coal mines, traditional laser beam image segmentation and feature extraction algorithms have some problems such as feature omission, extraction error, and extraction difficulty in underground laser beam image extraction. It is necessary to further study the stable and accurate segmentation and extraction method of laser beam target images that are suitable for complex underground environments, so as to further improve the performance of the laser beam target-based long-distance vision positioning system. The laser beam target-based visual positioning system was tested industrially at the working face, and the laser beam images acquired are shown in Figure 1.

The line feature of the laser beam target is the visible laser beam produced by the Tyndall effect of the laser beam due to the dust and water fog in the mine. The laser beam-based target is formed by the laser beams emitted by the laser pointing instrument that was installed above the tunneling roadway. Line feature segmentation and extraction are the keys to the precise visual positioning of a boom-type roadheader by using an artificial laser beam target. Due to the complex environment of the tunneling face, the line feature information of the laser beam target used for pose calculation is easily confused by the background, especially when the distance between the vision sensor and the laser pointing instrument increases, whereby the laser beam target information will be weak, resulting in difficult feature extraction. Therefore, a laser beam image segmentation and feature extraction network model is a necessity to provide the data basis for the accurate and real-time visual positioning of tunneling equipment. At the same time, in order to solve the problem that the laser beam features are not obvious when the dust concentration is low, the laser beam feature enhancement needs to be adopted in the process of laser beam image segmentation and feature extraction to ensure the stability of laser beam feature extraction.

Aiming at the problem that laser line features are difficult to extract in the complex environment of a coal mine, a multi-laser-beam target image segmentation network model is needed to realize the laser beam target feature segmentation and extraction, and to obtain the center-line information. At present, the advantage of the traditional image segmentation method is that its segmentation performance in a single background is more stable, but for complex scenes such as underground coal mines, the robustness of the traditional algorithms cannot achieve the expected results [4]. With the rise of computer vision technology, image semantic segmentation technology based on deep learning has made significant progress, and high segmentation stability can be achieved by using specific network models in specific scenes. Aiming at harsh environments such as high dust, high water mist, and uneven light at the tunneling face of underground coal mines, and combined with the distribution characteristics of the laser beam target, this work builds a multi-laser-beam target image semantic segmentation network RCEAU-Net for the complex environment of a coal mine tunneling face based on the traditional U-Net, inspired by the encoder–decoder structure of the U-Net network, aiming to provide accurate and real-time laser beam features for the visual positioning of the boom-type roadheader. The main contributions of this paper are as follows:

- Aiming at the problem of multi-laser-beam segmentation and extraction faced by the remote distance vision positioning system in underground application, an RCEAU-Net model suitable for the laser beam image segmentation in the underground working face is proposed. The reliable segmentation and accurate extraction of laser beam features are realized under the condition of complex background interference, distance change, and constant change in dust concentration in the coal mine.
- The proposed RCEAU-Net model effectively fuses the underlying feature information by introducing residual connections in the convolution of the encoder and decoder structures of U-Net. Meanwhile, the problem of missing contextual semantic information in U-Net is compensated by introducing cascade multi-scale convolution in the skipping connection part. In addition, the introduction of an efficient multi-scale attention module with cross-spatial learning in the encoder enhances the feature extraction ability of the network for laser beams, which improves the segmentation effect of the network model on tiny laser beams at long distance.
- The LBTD was constructed with images collected from multiple scenarios of different distances, dust concentrations, low illumination, and overexposure in coal mines. Based on LBTD datasets, the experiment was carried out for the validation of the image segmentation performance of the constructed RCEAU-Net. The results demonstrated that the proposed RCEAU-Net realizes the accurate and reliable segmentation of the boundary features and tiny features of laser beam images in the complex background, and it can meet the demand for laser beam segmentation for long-distance visual localization in coal mines.

The rest of this paper is organized as follows, Section 2 introduces some related work within this topic; Section 3 gives an overview of the traditional U-Net network, based on which the RCEAU-Net semantic segmentation network proposed in this paper is described in detail, and summarizes the method for feature enhancement of red laser beams under the complex conditions of the coal mine tunneling face, and describes the scheme for constructing the LBTD of the laser beam segmentation dataset; the construction of the related experimental platform as well as the experimental demonstration is given in Section 4; and Section 5 discusses and concludes the research work of this paper.

## 2. Related Work

Multi-laser-beam target feature extraction accuracy mainly depends on the stability and robustness of the image segmentation method; at present, image segmentation methods can be divided into traditional image segmentation methods and deep learning-based image segmentation methods. Traditional image semantic segmentation methods mainly use pixel color information, gradient histogram information, grey scale information, edge information, and other features of the image to complete the task of image segmentation in different scenes [5]. Before the deep learning-based image semantic segmentation network was proposed, traditional image segmentation methods were applied in the fields of road sign extraction [6], medical image segmentation [7], plant disease image segmentation [8], etc., which shows that the traditional algorithms have good application feasibility in simple scenes.

With the application and development of computer technology, the requirements of industrial intelligence are getting higher and higher, and the demand for image segmentation tasks under complex backgrounds is rising. Constrained by the traditional algorithm design difficulties, whereby it is difficult to ensure real-time target segmentation in the complex background of poor results and other issues, researchers have begun to choose to use deep learning-based image semantic segmentation methods to build segmentation models to complete the target segmentation task. Currently, the design of segmentation models based on the encoder–decoder structure of a full convolutional neural network FCN [9] is quite extensive, among which, due to the relatively simple structure of the U-Net [10] model and its outstanding segmentation performance, it and its variants have now achieved remarkable results in the semantic segmentation tasks of images such as medicine [11], traffic [12], agriculture [13], aerial photography [14], remote sensing [15], and so on. O. Oktay et al. [16] proposed a novel Attention Gate (AG) model for the medical image domain, which can automatically learn to focus on target structures of different shapes and sizes, and integrated it into the U-Net network architecture to build the Attention U-Net network, which reduces the computational overheads of the original U-Net model, and improves the model’s sensitivity and computational accuracy. To bridge the feature mapping gap between sub-networks caused by the original U-Net encoder–decoder architecture, Zhou, Z et al. [17] proposed a more efficient medical segmentation architecture, U-Net++, which utilizes a deeply supervised encoder–decoder network by re-designing the skipping connections of the U-Net network to partially improve the medical image segmentation results. Foivos I. Diakogiannis et al. [18] proposed a ResUnet network by combining the U-Net network with the residual structure, which improves the accuracy of semantic segmentation task for high-resolution aerial images, and solves the problem of gradient vanishing and exploding, which exists when the network is structured in a deeper way. Huang H et al. [19] addressed the lack of full-scale semantic information transfer in a U-Net++ network, introduced full-scale skipping connections between the encoder and decoder to combine low-level semantic information with high-level semantic information from other feature maps of different scales, established a U-Net3+ model, and achieved better results in organ segmentation task, and also, the design of the model reduces the network parameters, which improves the computational efficiency. Aiming at the problem that convolutional operations cannot satisfy the global semantic information transfer, H Cao et al. [20] proposed a Swin-Unet network architecture, in which a shift-window-based hierarchical Swin Transformer is used as an encoder to extract contextual features, and a Swin Transformer-based patch extended layer symmetric decoder is designed to achieve up-sampling. At the same time, skipping connections were used to learn local–global features, and the model achieved better results in organ and heart segmentation tasks. Li C et al. [21] designed a nested attention-aware segmentation network model, Attention unet++, to improve the effectiveness of the resection of necrotic portions of the liver, which incorporates the deep supervised encoder–decoder architecture in the U-Net++ structure as well as dense skipping connections, and introduced an attention mechanism between nested convolutional blocks, and validated the model for automatic segmentation of necrotic liver on the MICCAI 2017 Liver Tumor Segmentation (LiTS) dataset. In order to solve the model degradation problem of U-Net++, Li Z et al. [22] proposed a Residual-Attention UNet++ network, which introduced the residual unit in U-Net++, and at the same time, added an attention mechanism to the network structure to enhance the weight of the segmentation target part and inhibit the background region that is irrelevant to the segmentation task, and validated it experimentally on the skin cancer, nucleus, and coronary angiography medical image sets for experimental validation, and better segmentation results were obtained. In order to detect road cracks in time and improve the safety of road traffic, an ARD-Unet network was proposed by Gao Y et al. [23] using UAV remote sensing images as a starting point, which combines a depth-separable residual block (DR-Block) based on U-Net, an Atrous Space Pyramid Fusion Attention Module (ASAM) and Receptive Field Block (RFB), which compensates for the problem of the loss of semantic feature information in the traditional U-Net, and improves the segmentation effect of road cracks in remote sensing images. In order to achieve automatic planning of transmission lines, Nan, G et al. [24] proposed an AS-Unet++ network to complete the accurate segmentation of remote sensing image features, which added the spatial pyramid pool (ASPP) and squeeze-and-excitation (SE) modules based on the traditional U-Net network to expand the model’s receptive field and enhance the feature information of the targets to be segmented. At the same time, combined with the skipping connection part in the structure of U-Net++, the feature extraction part of each layer is stacked, which makes up for the problem of semantic feature loss caused by the traditional skipping connection; compared with U-Net, AS-Unet++ has a significant increase in the segmentation accuracy of remote sensing images. Li, Y. et al. [25] proposed a U-Net citrus plantation extraction model based on an image pyramid structure to accurately extract citrus plantation areas based on Sentinel-2 satellite images, using the pyramid structure encoder to capture contextual information at multiple scales, and using spatial pyramid pooling to prevent information loss and improve the ability to learn spatial features, which achieves high-precision large-scale citrus plantation segmentation. Khan, M.A.-M. et al. [26] proposed a Dense U-Net network to segment cracks on railway sleepers based on the U-Net network model in response to the time-consuming and inefficient traditional methods of detecting cracks on railway sleepers. In this model, several short connections are established between the encoder and decoder modules of the original U-Net network, so that more semantic information is obtained in the skipping connection part of the network, and the segmentation accuracy of railway crack images is improved.

With the wide use of U-Net, researchers in the field of coal mining have also begun to apply U-Net to some related tasks. Inspired by the excellent performance of neural networks in the field of image segmentation, Houxin Jin et al. [27] proposed an M2AR-U-Net segmentation model for coal rock feature extraction, which achieves the accurate segmentation of coal rocks from the background region. In order to investigate the particle characteristics of tar-rich coal mines before separation, Jinwen Fan et al. [28] combined the image segmentation model of the U-Net variant with OpenCV feature extraction, and systematically analyzed the particle morphology, particle size, release characteristics, and density separation process of coal mines. Fengli Lu et al. [29] proposed a deep neural network, A-DNNet, based on the U-Net framework to extract micro-cracks from coal rock images in continuous frames. Experiments show that A-DNNet can achieve more stable and efficient segmentation of sequential coal rock cracks compared with the original U-Net algorithm. In order to overcome the problems of mis-segmentation and omission of segmentation of traditional methods in the coal processing industry, Yihao Fu et al. [30] proposed a U-network based on simple linear iterative clustering (SLIC) for superpixel preprocessing, and compared it with the traditional watershed algorithm, and obtained more accurate segmentation results.

In summary, since U-Net was proposed, its good segmentation performance, as well as small-sample training advantage, has received the favor of many researchers, and its explosive growth in various structures has explored the great potential of U-Net networks [31] so that semantic segmentation methods based on U-Net and its variants have matured and been applied in most fields, but due to the complex conditions of the tunneling face of underground coal mines such as high dust, high water mist, high noise, and uneven illumination, and other problems such as laser beam feature information that is not obvious and easy to confuse with the background, the existing model is not sufficient to solve the task of the segmentation of the laser beam target feature in coal mine undergrounds. Therefore, based on this previous research, this study establishes the RCEAU-Net framework based on the encoder–decoder structure of U-Net by introducing the residual unit, cascade multi-scale convolution (CMSC) module, and the Efficient Multi-Scale Attention Module with Cross-Spatial Learning (EMA). The specific details of the network architecture will be detailed in the following Sections.

## 3. Methods

### 3.1. The U-Net Network Overview

**Figure 2:**
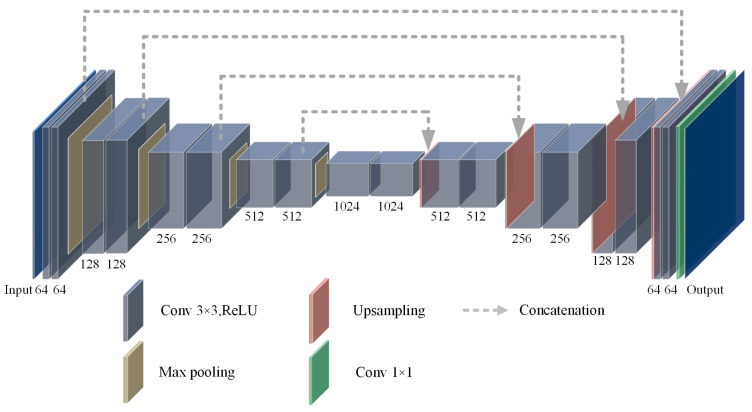
The architecture of the U-Net network model.

The U-Net network consists of three main components: encoder, decoder, and skipping connections. Among them, the encoder consists of multiple convolutional blocks and maximum pooling module, which serves for extracting input image features. Where each convolution block contains a convolution operation and ReLU activation followed by a maximum pooling operation to reduce the resolution of the feature map. The decoder maps the feature maps extracted by the encoder back to the original input image space by means of multiple up-sampling modules and convolutional blocks. The skipping connection part is the key design for U-Net network to be able to segment the target accurately, which achieves the feature information transfer by directly connecting the corresponding feature maps of the encoder and the decoder. After each encoder down-sampling, the skipping connection retains the corresponding layer of feature maps and connects with the up-sampled feature maps on the decoder path to achieve the integration of feature information. This design avoids the loss of semantic information to a certain extent, enhances the network’s ability to perceive the details and local features, and effectively improves the robustness of the U-Net in the task of image segmentation. The architecture of the U-Net is shown in Figure 2.

The feature fusion strategy adopted by U-Net is very representative, and its U-shaped structure and skipping connection design between the encoder and the decoder can achieve good feature information transfer, effectively avoiding the problem of information loss, and, at the same time, the structure ensures that U-Net can adapt to different sizes of the input image, with good generalization ability. The underground environment of a coal mine is complex and the data are difficult to obtain, while U-Net performs well in small-sample learning and can achieve good results even in the case of limited data. Therefore, this paper constructs the RCEAU-Net network based on the U-Net network architecture, combined with the characteristics of laser target images in underground coal mines.

### 3.2. RCEAU-Net

Although the traditional U-Net is able to achieve accurate segmentation in different fields, there are still some problems in the laser beam target segmentation task for the laser beam marking in the face of the underground digging workings in coal mines. Specifically, the skipping connection part of the U-Net fails to effectively transfer the information between low-level features and high-level features, resulting in the lack of multi-scale feature information, which affects the extraction effect of the features on the boundary and detailed parts of the downhole laser beam. In addition, when the concentration of work-face cut-off dust decreases, the laser beam emitted by the downhole laser point instrument produces limited feature information due to the restricted Tyndall effect, which is easily confused with the complex background of the downhole, thus leading to the segmentation model, and problems such as omission and misjudgment.

Due to the environmental characteristics such as high noise and uneven illumination at the tunneling face of the coal mine, the laser beams acquired at long distances are weak and the target area is small, and it is difficult to distinguish them from the background. Although the skipping connection part of the traditional U-Net can help to fuse different levels of features, its ability to process multi-scale information is relatively limited, and the simple splicing or summing operation cannot adapt to the feature extraction of tiny targets, which leads to the failure of laser beam segmentation at long distances and when the concentration of cut-off dust is low.

**Figure 3:**
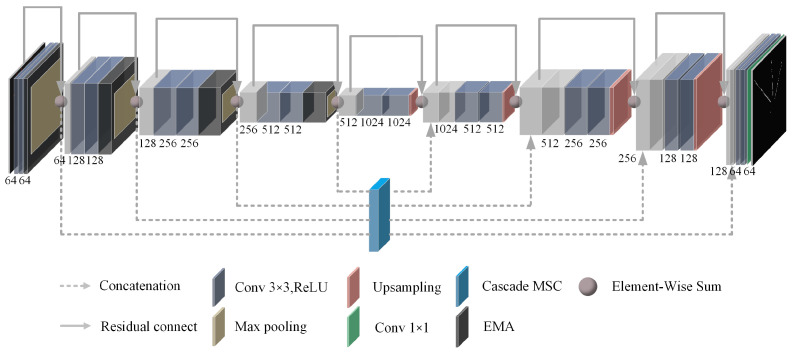
The architectures of the RCEAU-Net.

Therefore, to address the above problems, we proposed the RCEAU-Net based on the U-Net architecture to complete the task of laser beam target segmentation for the laser beam marking feature at the face of an underground coal mine. The architectures of the RCEAU-Net network are shown in Figure 3. Among them, the design of the Residual structure (R), Cascade Multi-Scale Convolution Module (C), Efficient Multi-Scale Attention Module with Cross-Spatial Learning (EA), and loss function are described in detail in Section 3.2.1, Section 3.2.2, Section 3.2.3 and Section 3.2.4, respectively.

#### 3.2.1. Residual Structure

In order to enhance the generalization ability of the U-Net segmentation network, better transfer and effectively use the underlying feature information, and improve the model’s multi-scale perception and characterization performance for laser beam features, we combined the residual structure in ResNet, and used the idea of residual connection in both the encoder and decoder parts of U-Net, respectively, using two 3 × 3 convolutions to form a residual block to replace the convolution block in traditional U-Net, in order to construct the encoder–decoder framework based on the residual structure. Among them, each convolutional block contains a BN layer, a ReLU activation layer, and a convolutional layer. Eventually, a feature encoder was built from four layers of convolutional blocks based on the obtained residual structure. Meanwhile, the corresponding decoder is symmetrically obtained based on the U-Net architectural properties for RCEAU network architecture construction.

**Figure 4:**
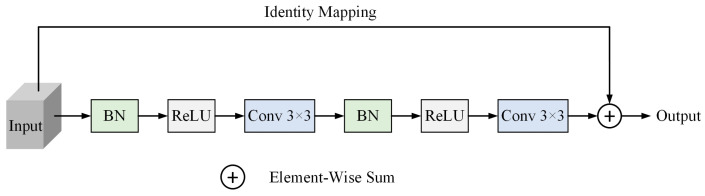
The residual block. Here, two sets of BN, ReLU, and Conv 3 × 3 are used for feature extraction of the input feature, respectively, and an identity mapping of the initial feature to the final feature is performed in place of the convolutional blocks in the conventional U-Net network.

The introduction of the residual structure not only enables the model to effectively fuse the underlying feature information, but also improves the gradient flow performance of the network and reduces the gradient vanishing problem during the training process. The residual connection improves the training stability and convergence speed of the network, enhances the processing capability of the model for the laser beam feature segmentation task in the complex environment of underground coal mines, better captures the boundary and detailed information of the laser beam, and improves the segmentation accuracy of the laser beam. In this paper, the model is named RU-Net. The residual block structure is shown in Figure 4.

#### 3.2.2. Cascade Multi-Scale Convolution Module

In order to solve the problem of the original U-Net network’s insufficient segmentation ability for tiny laser beams when the visual sensors are far away from the laser point instrument, this paper introduces the CMSC module in the skipping connection part, which aims to make up for the original U-Net network’s lack of contextual semantic information in the skipping connection part, and thus improve the model’s segmentation effect for tiny laser beams. CMSC captures multi-scale features by introducing multiple parallel convolutional layers and making full use of the different receptive fields of each convolutional layer. Through scale feature fusion, a rich feature representation with multi-scale is formed, which successfully preserves the details and global information in the image.

**Figure 5:**
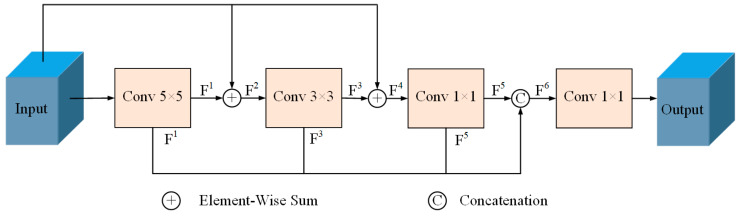
The CMSC module. Here, three kinds of convolution kernels, Conv 5 × 5, Conv 3 × 3, and Conv 1 × 1, are used to build a cascade multi-scale convolution module to extract multi-scale feature information, and complete the fusion of feature maps through “Concatenation”, and finally use Conv 1 × 1 to restore the feature dimensions, so as to make up for the semantic gaps in the part of the jump connection.

In this paper, the CMSC is built with three convolution kernels, Conv 5 × 5, Conv 3 × 3, and Conv 1 × 1. First, the input feature map is subjected to the Conv 5 × 5 operation to obtain a more global feature map, which is noted as F^1^. Then, the original feature map is used to superimpose with F^1^ to obtain the feature map F^2^. Subsequently, Conv 3 × 3 is used to capture F^2^ to obtain the feature map F^3^. F^3^ is superimposed with the original feature map to extract the richer features, which is noted as F^4^. And then, Conv 1 × 1 is used to capture F^4^ and obtain the feature map F^5^. Finally, F^1^, F^3^, and F^5^ are fused at multiple scales to obtain a multi-scale feature map, denoted as F^6^. The abstract and detailed feature information retained by F^6^ at different scales at the same time improves the segmentation capability of the model for weak laser beams, bridges the semantic gap in the traditional U-Net skipping connection, and is capable of better adapting to the size change in laser beams at different distances; this design makes the model more adaptable to the scenarios that deal with the segmentation task requirements of tiny laser beams, and effectively improves the segmentation effect of the model. The CMSC module is shown in Figure 5.

#### 3.2.3. Efficient Multi-Scale Attention Module with Cross-Spatial Learning

Due to the complex features of the underground coal mine environment, which contains various noises such as stray light, dust, water mist, etc., these factors affect the stability of the network model in the laser beam target segmentation task. In order to enhance the encoder’s ability to extract laser beam features from underground coal mines and suppress the network’s extraction of irrelevant features such as noise, this manuscript introduces an Efficient Multi-Scale Attention Module with Cross-Spatial Learning [32] after each residual structure-based convolutional block of the encoder portion of RU-Net to strengthen the network’s segmentation performance for laser beam features.

Compared with Convolutional block attention module (CBAM) [33], Normalization-based Attention Module (NAM) [34], Shuffle attention (SA) [35], Efficient channel attention (ECA) [36], and Coordinate attention (CA) [37] attention mechanisms, the Efficient Multi-Scale Attention Module (EMA) can reduce the computational overhead while efficiently and stably attending to and utilizing the different channel information and spatial information of the input features, enabling the model to improve the attention of the target features.

When the traditional attention module performs sequential computation, it leads to a large network depth and complex model computation. When the convolution operation is carried out in the module, it often leads to a reduction in the feature map channel dimension, thus failing to effectively characterize the channel dimension information, and failing to produce better pixel-level attention for the mapping of higher-order features. Therefore, in order to solve the module complexity caused by sequential computation and the channel dimension reduction problem caused by convolution, the EMA attention mechanism slices the input feature map $X\in I^{C\times H\times W}$ into G sub-feature maps in the channel dimension C, i.e., $X_{i}\in I^{G//C\times H\times W}$ is obtained; then, at this point, $X=[X_{0},X_{i},\ldots ,X_{G-1}]$, which is used for the subsequent weight computation, where C>>G. Based on this, EMA designed a parallel branching structure to compute and fuse the grouped feature maps through two different branches to improve the target region weights. Among them, the two branches are the 1 × 1 branch formed by the shared component of the 1 × 1 convolution extracted from the coordinate attention (CA) and the newly designed 3 × 3 convolution kernel branch.

**Figure 6:**
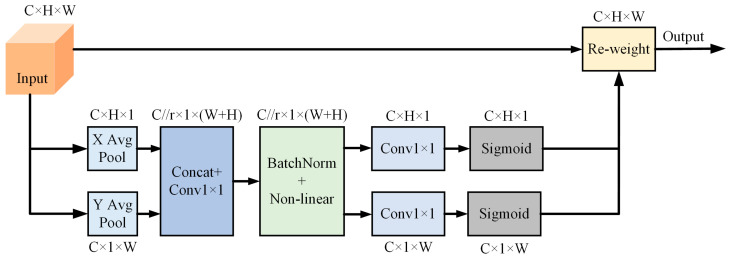
CA module. “C”, “H”, and “W” are the number of channels, height, and width of the feature maps, respectively. “X Avg Pool” refers to 1D horizontal global pooling, and “Y Avg Pool” means 1D vertical global pooling. “Re-weight” is the adjusted weight matrix.

The shared component of the 1 × 1 convolution extracted from the CA attention module is used to accurately embed the location information in the channel information and to achieve remote interaction in spatial location, which enables the convolution operation to learn effective channel information without reducing the channel dimensions in order to accurately embed the spatial location information in the channel attention. Specifically, the branch designs two one-dimensional global average pooling layers along the two spatial dimensions X and Y, respectively, encodes the global information through global average pooling, and compresses the global spatial location information into the channel attention graph, which enhances the feature fusion between the channel and the spatial information. The CA Attention module is shown in Figure 6.

In order to ensure that the whole spatial locations can interact with each other and fuse the spatial feature information at multiple scales, EMA computes the 1 × 1 branch of CA in parallel in the X and Y spatial dimensions, respectively, and at the same time, places the two paths in parallel with the 3 × 3 branch to obtain a total of three computational paths. In the 1 × 1 branch, when the input feature map is subjected to the average pooling operation, the output is decomposed into two vectors using the shared 1 × 1 convolution, and then two Sigmoid nonlinear functions are used to fit the convolution output results, and finally, the information of the channel attention maps obtained by the two paths of the 1 × 1 branch is fused using the multiplication method. In the 3 × 3 branch, a 3 × 3 convolution kernel is used to capture the multi-scale information of the feature maps in order to enhance the local cross-channel information interaction and facilitate the fusion of contextual information at different scales. Through the above cross-channel information interaction and modeling, EMA effectively establishes the importance distribution of different channels, while being able to stably combine spatial and channel information.

**Figure 7:**
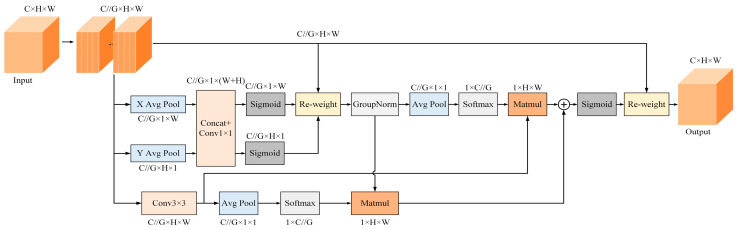
EMA module. “G” means grouping, “X Avg Pool” denotes the one-dimensional horizontal global pooling, and “Y Avg Pool” means one-dimensional vertical global pooling.

In order to better characterize and fuse richer features, EMA utilizes a cross-space fusion approach in different spatial dimensional directions to learn features for each computational path and enhance the interactions across latitudes. Firstly, global average pooling is used to encode the global spatial information on the outputs of the two branches of 1 × 1 and 3 × 3 respectively, and a Softmax natural nonlinear function is fitted to them, and the outputs on each branch are multiplied by the matrix dot product in order to obtain the two respective spatial attention maps. Then, the output features of each grouping are mapped using the set of the two spatial attention weight matrices and the Sigmoid activation function to obtain pixel-level correspondences and complete the cross-space learning. For the laser beam target segmentation task, EMA can effectively adjust the target position weights and improve the model segmentation performance while ensuring real-time performance. The module of EMA is shown in Figure 7.

#### 3.2.4. Loss Function

The loss function is used to measure the difference between the predicted output of the segmentation model and the true labels, which can guide the network training to make the model’s prediction closer to the true labels and evaluate the model’s performance on the validation set or test set. In U-Net networks, the training of segmentation models is generally completed using the binary cross-entropy loss function (BCELoss), which motivates the models to focus more on the accuracy of the segmentation results. However, BCELoss only takes into account the information between pixel levels; in order to ensure that the model has a better discriminative ability for the global features of the laser beam and its boundary position during the training process, this paper introduces the loss function part of PraNet [38], which combines the intersection and concatenation ratio loss (IoULoss) in addition to the BCELoss, so that the loss function can quantify the difference between the segmentation results of the laser beam in the global features and the real label, pay more attention to the integrity of the laser beam segmentation, improve the model’s attention to the laser beam boundary, optimize the effect of the laser beam detail segmentation, and further improve the detection performance of the tiny laser beam. The formulas for BCELoss and IoULoss are shown in Equations (1) and (2), respectively.

(1) $$L_{BCE}=-\sum_{\left(r,c\right)}\left[G(r,c)log(S(r,c))+(1-G(r,c))log(1-S(r,c))\right],$$

(2) $$L_{IoU}=1-\frac{\sum_{r=1}^{H}\sum_{c=1}^{W}S(r,c)G(r,c)}{\sum_{r=1}^{H}\sum_{c=1}^{W}\left[S(r,c)+G(r,c)-S(r,c)G(r,c)\right]}$$

where G(r,c)∈{0,1} is the true label at pixel point (r,c), and S(r,c) is the predicted probability of the target.

In order to enhance the flexibility of the loss function, in the design of the loss function, the traditional loss calculation method with uniform global weights is discarded, and the weighted approach is used to complete the value calculation for BCELoss and IoULoss, respectively. The weighted design enables the model to better focus on the laser beam region in the image and reduce the impact of data noise on the model. In this paper, we use the difference obtained by subtracting the result of the binary label map after average pooling from itself to adaptively measure the relative importance of the pixels in the image, when the difference is large, it means that there is a significant difference between the labeled image and the average pooled map, which indicates that the computed region contains more difficult to capture the information, and it should be given stronger attention; on the contrary, when the difference is smaller, it means that the computed region is relatively consistent in terms of its content, which has less impact on the segmentation task and should be given less attention; in this paper, the difference obtained by subtracting the average pooled binary labeled image from the original binary labeled image is used as the dynamic weight ω to emphasize the importance of the laser beam boundaries and to suppress the attention of irrelevant regions.

The weighted sum of BCELoss and IoULoss using the obtained weight matrix ω completes the construction of the final overall loss function, which alleviates the influence of noise on the loss function, further enhances the focus of the segmentation model on the laser beam boundary, and improves the segmentation performance of the model on the weak laser edges. The final obtained overall loss function of RCEAU-Net is defined as shown in Equations (3) and (4). In this paper, this loss function is denoted as StructLoss.

(3) $$L_{total}=L_{BCE}^{\omega }+L_{IoU}^{\omega }$$

(4) $$\omega =X-X_{pool}$$

where $L_{total}$ is the overall loss function, $L_{BCE}^{\omega }$ is the weighted BCELoss, and $L_{IoU}^{\omega }$ is the weighted IoULoss. ω is the weight matrix, X is the actual labeled map of the laser beam, and $X_{pool}$ is the global average pooled graph over X.

### 3.3. Underground Coal Mine Laser Beam Target Dataset

In this paper, the multi-laser-beam target image data are collected from the tunneling working face of several coal mines. Due to the complexity of the working environment of coal mines, the collected laser beam images have a diversity of coal dust concentration change, low dust, high dust, low illumination, overexposure, distance change, etc. The laser beam image acquisition is made with an industrial camera of MV-EM510C (HD514-MP2), with a lens resolution of 2048 × 2456, and a focal length of 5 mm. The wavelength of the mining laser pointing instrument is 658 nm, the advantage of this wavelength is that it is able to produce a good Tyndall effect in dust, and can enhance the visibility of the laser beam in the image through the color component constraints.

**Figure 8:**
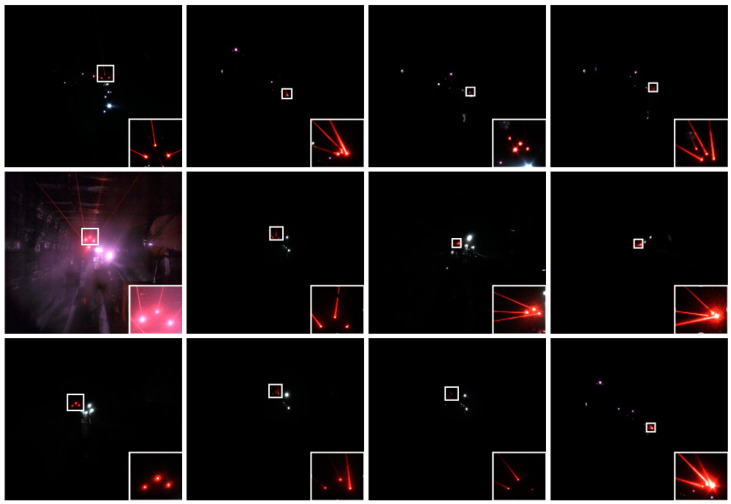
The Laser beam target images. The bottom right area shows a local enlargement of the laser beam target in the original image.

The laser beam image data were obtained by collecting the laser beams above the roadway behind the fuselage by the camera installed on the fuselage of the boom-type roadheader. The distance range between the camera and the mining laser pointing instrument was about 10 m~80 m. Due to the complexity of the working conditions in underground coal mines, the acquired image data contain different distances, different dust concentrations, and different lighting conditions and other distribution types. Meanwhile, because a large number of similar frames are generated in the process of image acquisition, the training will reduce the generalization of the model, resulting in the overfitting phenomenon. Therefore, we built an automatic filtering network based on ResNet50 to filter the acquired raw data [39], aiming to eliminate similar images, images that are heavily occluded, or where the laser beam target features are missing, that making it impossible to perform annotation. Meanwhile, in order to further ensure the effectiveness of data filtering, manual screening was used to check the filtered data, and the final 3406 images were used as our image data for the laser beam target dataset production. Some of the obtained image data are shown in Figure 8.

The laser beams emitted by the mining laser point instrument rely on the diffusion of the cutting dust from the roadheader, by using the Tyndall effect to complete the visible laser beam imaging in the industrial camera. Therefore, the cutting dust concentration will directly affect the quality of the laser beam features in the collected image. When the cutting is stopped at the tunneling face, the dust concentration in the environment decreases, and it is difficult for the laser beams emitted by the laser point instrument to produce a strong Tyndall effect, which leads to a reduction in the visible laser beam features under the camera imaging, and it makes the manual annotation production and laser beam target segmentation more difficult. Therefore, by combining the environmental characteristics of the tunneling face in underground coal mines and the wavelength of the laser point instrument used in this work, a red laser beam feature enhancement module is proposed in this Section to improve the quality and efficiency of manual annotation production. Meanwhile, it is used as an image preprocessing module before network segmentation to enhance the visible features of the red laser beams and the segmentation stability of the network. The judgment condition depends on the color component distribution constraint between the R, G, and B channels of the laser beam image, and the part of each channel that does not satisfy the condition is assigned to 0, and the rest of the values are kept unchanged in order to perform noise filtering. Combined with the laser beam characteristics of the 658 nm wavelength, this paper shows through the image of the same pixel under the R channel and the remaining two channels that the difference is greater than 0, as a judgment condition for noise filtering; if greater than 0, it means that this is a laser beam target feature, if less than 0, then this is a noise point.

**Figure 9:**
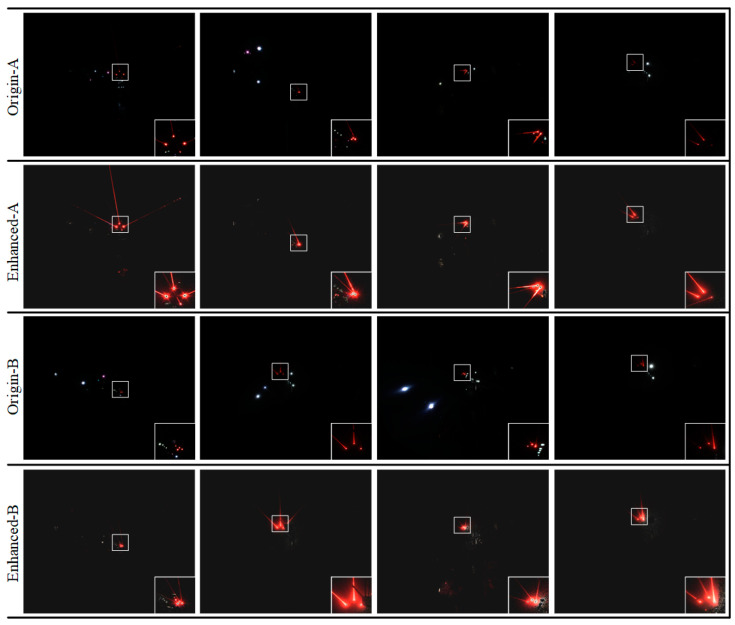
Laser beam image feature enhancement. “Origin” indicates the original image captured with the industrial camera. “Enhanced” indicates the enhanced image.

Subsequently, each channel after conditional judgment is median-filtered to reduce the influence of noise on the image quality, and the filtered three channels are merged to obtain the filtered RGB map. Adaptive histogram equalization is used to adjust its brightness and contrast to obtain the laser beam feature-enhanced image. Parts of the enhanced images are shown in Figure 9.

The 3406 original images with manual screening were enhanced using the above laser beam feature enhancement method. The laser beam targets were manually annotated using the annotation software Labelme tool (5.3.1) (https://github.com/labelmeai/labelme, accessed on 10 September 2023), and during the annotation process, the images were annotated in accordance with the VOC dataset format to obtain visual labels in PNG file type. Subsequently, the image data and labeled data were both divided according to the ratio of 8:2, of which 2725 were used as the training set and 681 as the validation set. The constructed laser beam targets image dataset was named LBTD. In addition, in order to calculate the distribution of images across different conditions, three members with rich working experience in the coal mining field were selected to discriminate the distribution of the images in the LBTD datasets. Among these, there were 635 images of high dust concentration, 128 images of low dust concentration, and 2643 images of normal dust concentration when the images of the LBTD dataset were divided according to the dust concentration; and there were 101 images of strong light illumination, 846 images of low light illumination, and 2459 images of normal light illumination when the images of the LBTD dataset were divided according to the light intensity.

## 4. Experiments and Performance

### 4.1. Training Environment and Parameter Settings

In this study, Python3.8 was used as the main programming language, and the PyTorch 2.0.0 framework was used to complete the RCEAU-Net network construction. CUDA11.8 and cuDNN8.7.0 were used as deep learning acceleration modules to ensure that GPU acceleration was used during training. Meanwhile, the initial learning rate (lr0) was set to $1\times 10^{-5}$, and the final learning rate (lrf) was $1\times 10^{-6}$. The momentum was set to 0.9, and the was set to Adam, where β1 and β2 were respectively 0.9 and 0.999. Set the maximum number of training rounds epochs to 100, the weight decay coefficient (weight decay) to 0, the batch size to 4, and the image size to 2048 × 2456. Using Windows 10 system with 13th Gen Intel(R) Core(TM) i9-13900K CPU purchased in China and NVIDIA GeForce TUF-RTX4080-O16G-GAMING (which OEM from ASUS, Taipei, China) to complete the laser beam segmentation model training.

### 4.2. Experimental Results and Analysis

In order to verify the feasibility of RCEAU-Net on the laser beam segmentation task as well as its adaptability and generalization in the different coal mine tunneling faces, we adopted the image acquisition method mentioned in Section 3.3, and re-collected a total of 615 laser beam target images as the test set of LBTD using the industrial camera, which were collected at different distances under different environments in both the actual coal mine working scenes and the simulated tunnel environments in the laboratory. In the process of laser beam image acquisition in the laboratory of simulation underground roadway, the camera was installed in the tracked mobile robot and the smoke maker was used to simulate the cutting dust, at the same time as taking into account the low light environment and stray light interference such as mine lamps. The accuracy, precision, recall, and IoU of several more commonly used segmentation task indexes are used to quantify the extraction results to verify the robustness of the segmentation model, and their calculation formulas are shown in Equations (5)–(8), respectively.

(5) $$Acc=\frac{TP+TN}{TP+TN+FP+FN}$$

(6) $$Pre=\frac{TP}{TP+FP}$$

(7) $$Rec=\frac{TP}{TP+FN}$$

(8) $$IoU=\frac{TP}{TP+FP+FN}$$

**Figure 10:**
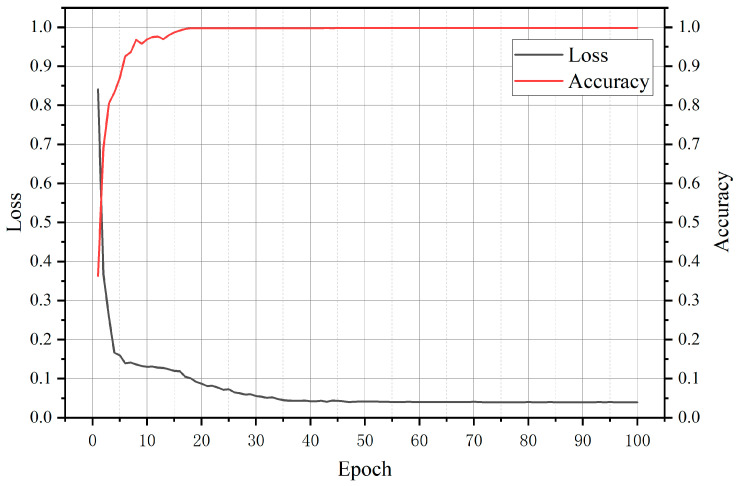
The loss and accuracy curves of RCEAU-Net when trained on LBTD datasets. The black curve represents loss and the red curve represents accuracy.

In the formula, *TP*, *TN*, *FP*, and *FN* represent the four indicators of the true-positive, true-negative, false-positive, and false-negative prediction results of the segmentation model, respectively. In this paper, *TP* represents the total number of downhole laser beam pixels correctly predicted by the model; *TN* represents the total number of downhole non-laser beam pixels correctly predicted by the model; *FP* represents the total number of non-downhole laser beam pixels predicted by the model as laser beam pixels; and *FN* represents the total number of downhole laser beam pixels predicted by the model as non-laser beam pixels. In this paper, we combine the above four quantitative metrics to build a network using five sets of improvement strategies using U-Net as a benchmark, and perform model evaluation on the LBTD test set to verify the effectiveness of each module. Among them, Improvement Strategy 1 is U-Net + StructLoss, Improvement Strategy 2 is RU-Net + StructLoss, Improvement Strategy 3 is RU-Net + StructLoss + EMA, Improvement Strategy 4 is RU-Net + StructLoss + CMSC, and Improvement Strategy 5 is RCEAU-Net. The improvement results of the network under different improvement strategies are shown in Table 1. It can be seen from the data in Table 1 that Improvement Strategy 5 achieves the highest score in all four groups of evaluation indexes, which is the optimal improvement scheme, proving the effectiveness and necessity of the introduction of each module. Although the introduction of the module increases the model inference time and training time, from the demand analysis of this study, for the feature extraction task of the laser beam target in an underground coal mine, the inference speed of the model is guaranteed to be within 80 ms per frame to meet the requirements. From the perspective of real-time inference, the inference time of RCEAU-Net is about 2.5 ms lower than that of the original U-Net, and its slightly longer inference time does not affect the real-time application of the model. The loss of RCEAU-Net when trained on LBTD and the trend of accuracy with the number of training rounds are shown in Figure 10.

As can be seen from Figure 10, the loss and accuracy curves of RCEAU-Net are able to converge stably and achieve high accuracy and small loss value, which shows that the laser beam segmentation model under RCEAU-Net has high detection accuracy and good fitting degree for the training data, and is able to stably complete the task of laser beam segmentation in an underground coal mine.

In order to further verify the segmentation effect of RCEAU-Net, we complete the training on LBTD using the four variants of Attention U-Net, U-Net3+, and Swin-Unet, which are the more popular U-Net network architectures at present, and Deeplabv3+ [40] semantic segmentation network, which is widely used in the industry at present, and test them on the test set. The performance evaluation of the different network models is obtained as shown in Table 2. The results of the assessment of the mean metrics under the different models are shown in Table 3. From the data in Table 3, it can be seen that RCEAU-Net is able to obtain better and more stable performance than U-Net and its more popular variants. In particular, accuracy, precision, recall, and IoU are improved by 0.19%, 2.53%, 22.01%, and 8.48%, respectively, over the traditional U-Net model.

**Figure 11:**
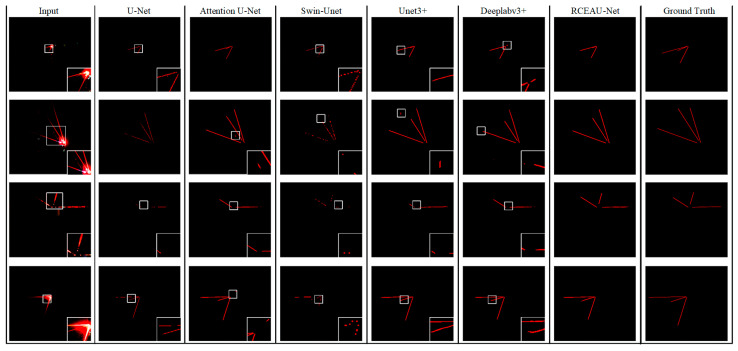
The segmentation effect of different models for laser beams.

In order to observe the segmentation results of the laser beam more intuitively, we visualized some of the segmentation results obtained by different models, and the results were collated as shown in Figure 11.

As can be seen from the figure, RCEAU-Net has a better segmentation effect for the laser beam in an underground coal mine; compared with other models, it not only has a better segmentation of the edge details of the laser line, but also reduces the mis-segmentation and omission of segmentation to a large extent, and it can well extract the weak visible laser beam under a low concentration of cut-off dust. To a certain extent, it meets the requirements of laser beam target feature segmentation in the underground working face and extraction, so as to ensure the stable and accurate positioning of tunneling equipment.

**Figure 12:**
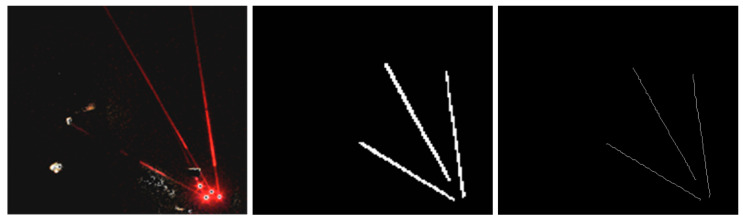
The feature refinement results.

In order to verify the effectiveness of RCEAU-Net on laser beam segmentation and feature extraction and to complete the pose calculation of the tunneling equipment based on the laser beam feature information, it is necessary to further obtain the slope and intercept of the laser beam center line. Here, the least squares method is adopted to carry out linear fitting of the laser beam center line obtained by segmentation. However, because the laser beam itself has a certain width, using the laser beam region to directly fit the straight line easily causes the problem of center-line deviation, resulting in the subsequent positioning accuracy. In order to ensure the linear fitting effect, an edge refinement algorithm is used based on the single-pixel edge mode [41] to refine the features of the segmented laser beam target to obtain finer laser beam features. The feature refinement results are shown in Figure 12.

**Figure 13:**
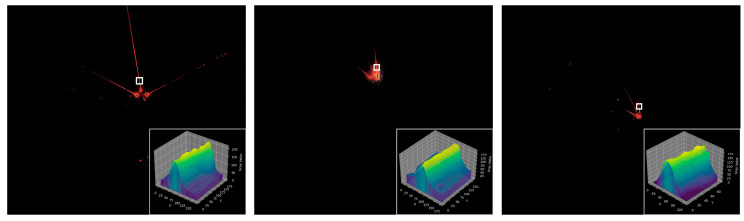
The 3D distribution of laser beam grey value, as shown in the lower right corner. The gray value of pixels close to the yellow region becomes larger, and the gray value of pixels close to the blue region becomes smaller.

Meanwhile, in order to improve the fitting effect of the straight line where the laser beam is located, so that each pixel point is as close as possible to the center line of the laser beam, this work analyzes the grey scale value of the center line of the laser beam and its surroundings. The distribution of grey scale values at the center line and its surroundings is shown in Figure 13.

**Figure 14:**
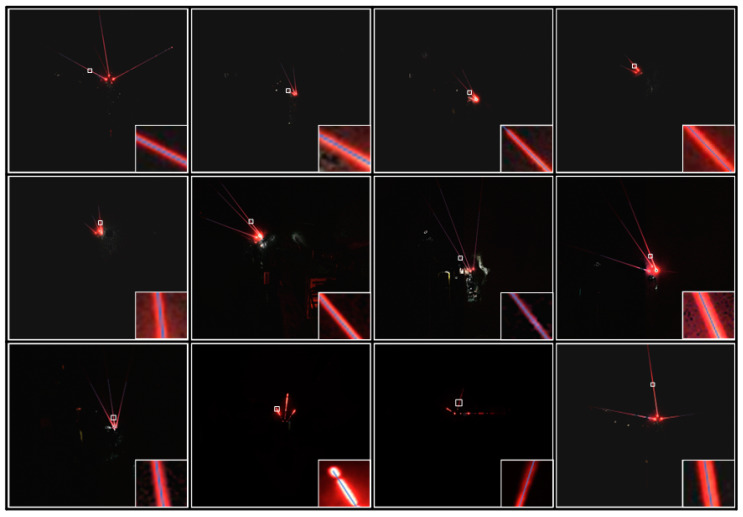
The center line fitting results of multi-laser beams.

From Figure 13, it can be seen that, in general, the grey distribution of the laser beams decreases sequentially from the center lines of the beam to both sides of the laser beams. Therefore, based on this grey scale distribution characteristic, this work establishes a 3 × 3 window and uses it to slide on the center line where the laser beam is located, and completes the corrective of the feature points that deviate from the spot center by selecting the grey scale maximum in each local window as the new feature point. The adoption of the local window is beneficial to accurately fit a straight line closer to the center of the laser beams, and to reduce the localization error brought by the straight-line deviation. The center line fitting results of multi-laser beams are shown in Figure 14.

From Figure 14, it can be concluded that by using RCEAU-Net to segment the laser beams to obtain the laser beam target area, and by fitting the center line after feature refinement through the least squares method, the slope and intercept information of the center line where the laser beam target is located can be obtained stably and efficiently, so as to provide stable straight-line features of the laser beam for realizing the precise positioning of the tunneling equipment. Even if part of the laser beam is blocked, the segmentation model will still have good performance. It can accurately complete the laser beam center-line extraction, and meet the requirements of the visual positioning model. In order to further verify the effectiveness of the center-line fitting, this work compares the partially fitted center-line information with the manually marked laser beam center-line information, and calculates the deviation of the slope and intercept between them. The parts of the comparison results are shown in Table 4.

From Table 3, it can be seen that the maximum deviation of the slope between the center line where the laser beam is located and the real center line obtained by fitting is 0.0085, and the maximum deviation of the intercept is 0.4306 pixels. We carried out a deviation analysis on a total of 615 sets of the laser beam center-line fitting results and obtained an average slope deviation of 0.0039 and an average intercept deviation of 0.2986 pixels, which can meet the requirements of the feature segmentation extraction of the center line where the laser beam is located in the complex background, and this is helpful to improve the visual positioning accuracy based on the laser beam target, and to complete the accurate pose measurement of the coal mine tunneling equipment.

## 5. Discussion and Conclusions

In this work, aiming at the problems of laser beam target image feature extraction and poor real-time performance faced by the long-distance visual localization system of tunneling equipment in the harsh environment of coal mines, we proposed a RCEAU-Net network model to segment the laser beam target features efficiently and stably. When the Tyndall effect is weak in the process of dust concentration change, the visible laser beam features generated are insufficient, which easily confuses the laser beam features with the background and makes it difficult to make artificial labels in the construction of the dataset. Laser beam feature enhancement is adopted to improve the visibility of the tiny laser beam under the weak Tyndall effect. At the same time, the laser beam images obtained by RCEAU-Net were refined and debiased, respectively, and the laser beam center line was obtained by the least squares method, which verified the effectiveness of RCEAU-Net and further ensured the accuracy and reliability of the visual localization for the tunneling equipment at the underground working face. The specific conclusions are as follows:

1. An RCEAU-Net model suitable for laser beam image segmentation in the working face is proposed for long-distance vision localization in an under-ground application. An LBTD was constructed with images collected from several different scenarios of a coal mine working face, which contains 3406 images and the laser beam target area that were manually labeled. The performance of the proposed RCEAU-Net model was significantly improved, and it can reliably segment and accurately extract laser beam features under the conditions of complex background interference, distance change, and coal dust concentration change.
2. Considering that it is difficult for traditional segmentation networks to obtain stable and accurate characteristics of multi-laser-beam targets, a new RCEAU-Net network is proposed in this work, which can effectively solve the problem of segmentation errors or omissions of laser beam target images due to weak laser beam features, discontinuity, and easy confusion with background. Moreover, although its inference speed is slightly slower than that of the U-Net network, the reasoning speed of the RCEAU-Net model can meet the requirement of real-time segmentation and extraction of downhole laser beam images.
3. The proposed underground laser beam segmentation network RCEAU-Net is verified on the established LBTD datasets. Compared with traditional U-Net, the accuracy is improved by 0.19%, precision is improved by 2.53%, recall is improved by 22.01%, and IoU is improved by 8.48%. The fitting accuracy of the laser beam center line is also verified and analyzed. The experimental results show that the maximum slope deviation between the fitted laser beam center line and the real center line is 0.0085, and the maximum intercept deviation is 0.4306 pixels, which can meet the accuracy requirements of laser beam feature extraction for long-distance visual localization in a coal mine.

From the above conclusions, it can be seen that the RCEAU-Net proposed in this manuscript is able to obtain good segmentation results under the premise of real-time performance for laser beam target segmentation under the complex background of the tunneling face in coal mines. However, when the industrial camera is affected by factors such as severe overexposure interference, excessive artificial occlusion, and severe motion blur, it will lead to the low quality of the captured image and result in bad results such as intermittent laser beam segmentation results and incorrect segmentation. Therefore, in our future work, we will focus on the problems of laser beam mis-segmentation and missed segmentation that are caused by camera overexposure, as well as camera motion blur, and concentrate on laser beam feature enhancement to improve the laser beam target segmentation effect in these cases.

## Figures and Tables

**Table 1:** Evaluation results under different improvement strategies.

No.	Network Model	mAcc	mPre	mRec	mIoU	Inference Time (per/ms)	Training Time per Epoch(s)
1	U-Net	0.9962	0.7091	0.6236	0.6014	5.6132	351
2	U-Net + StructLoss	0.9972	0.7115	0.8056	0.6707	5.5005	354
3	RU-Net + StructLoss	0.9974	0.7151	0.8196	0. 6724	5.9147	243
4	RU-Net + StructLoss + EMA	0.9979	0.7251	0.8360	0.6746	6.9504	334
5	RU-Net + StructLoss + CMSC	0.9978	0.7191	0.8383	0.6768	7.1446	363
6	RCEAU-Net	0.9981	0.7344	0.8437	0.6862	8.1003	430

**Table 2:** Evaluation results of laser beam target and background under different models.

No.	Network Model	Category	Acc	Pre	Rec	IoU
1	U-Net	Laser beam target	0.9945	0.5073	0.3459	0.2817
		Background	0.9979	0.9109	0.9013	0.9211
2	Attention U-Net	Laser beam target	0.9977	0.5247	0.7301	0.4236
		Background	0.9981	0.9121	0.9033	0.9238
3	Swin-Unet	Laser beam target	0.9950	0.4969	0.3239	0.2758
		Background	0.9982	0.9095	0.9089	0.9244
4	U-Net3+	Laser beam target	0.9970	0.5300	0.7665	0.4445
		Background	0.9986	0.9088	0.9101	0.9091
5	DeepLabv3+	Laser beam target	0.9972	0.5281	0.7458	0.4337
		Background	0.9980	0.9101	0.9132	0.9163
6	RCEAU-Net	Laser beam target	0.9979	0.5595	0.7767	0.4525
		Background	0.9983	0.9093	0.9107	0.9199

**Table 3:** Mean metrics evaluation results under different models.

No.	Network Model	mAcc	mPre	mRec	mIoU
1	U-Net	0.9962	0.7091	0.6236	0.6014
2	Attention U-Net	0.9979	0.7184	0.8167	0.6737
3	Swin-Unet	0.9966	0.7032	0.6164	0.6001
4	U-Net3+	0.9978	0.7194	0.8383	0.6768
5	DeepLabv3+	0.9976	0.7191	0.8295	0.6750
6	RCEAU-Net	0.9981	0.7344	0.8437	0.6862

**Table 4:** The comparison of center-line fitting results with ground truth.

No.	Slope			Intercept (Pixels)		
	GT	Fitting	Deviation	GT	Fitting	Deviation
1	0.5074	0.5075	−0.0001	210.5813	210.5532	0.0281
2	5.7172	5.7087	0.0085	−7036.5186	−7036.7213	0.2027
3	−0.4989	−0.5005	0.0016	1600.8748	1601.2083	−0.3335
4	0.5716	0.5743	−0.0027	312.6917	312.5921	0.0996
5	1.8137	1.8145	−0.0008	−1454.4894	−1454.1673	−0.3221
6	6.5202	6.5191	0.0011	−8169.6474	−8170.0015	0.3541
7	0.3314	0.3289	0.0025	674.7073	674.2767	0.4306
8	1.0353	1.0294	0.0059	−336.2056	−335.799	−0.4066
9	2.2483	2.2425	0.0058	−2075.6864	−2076.0135	0.3271

## Data Availability

Data are contained within the article.
